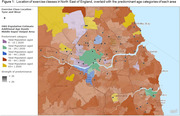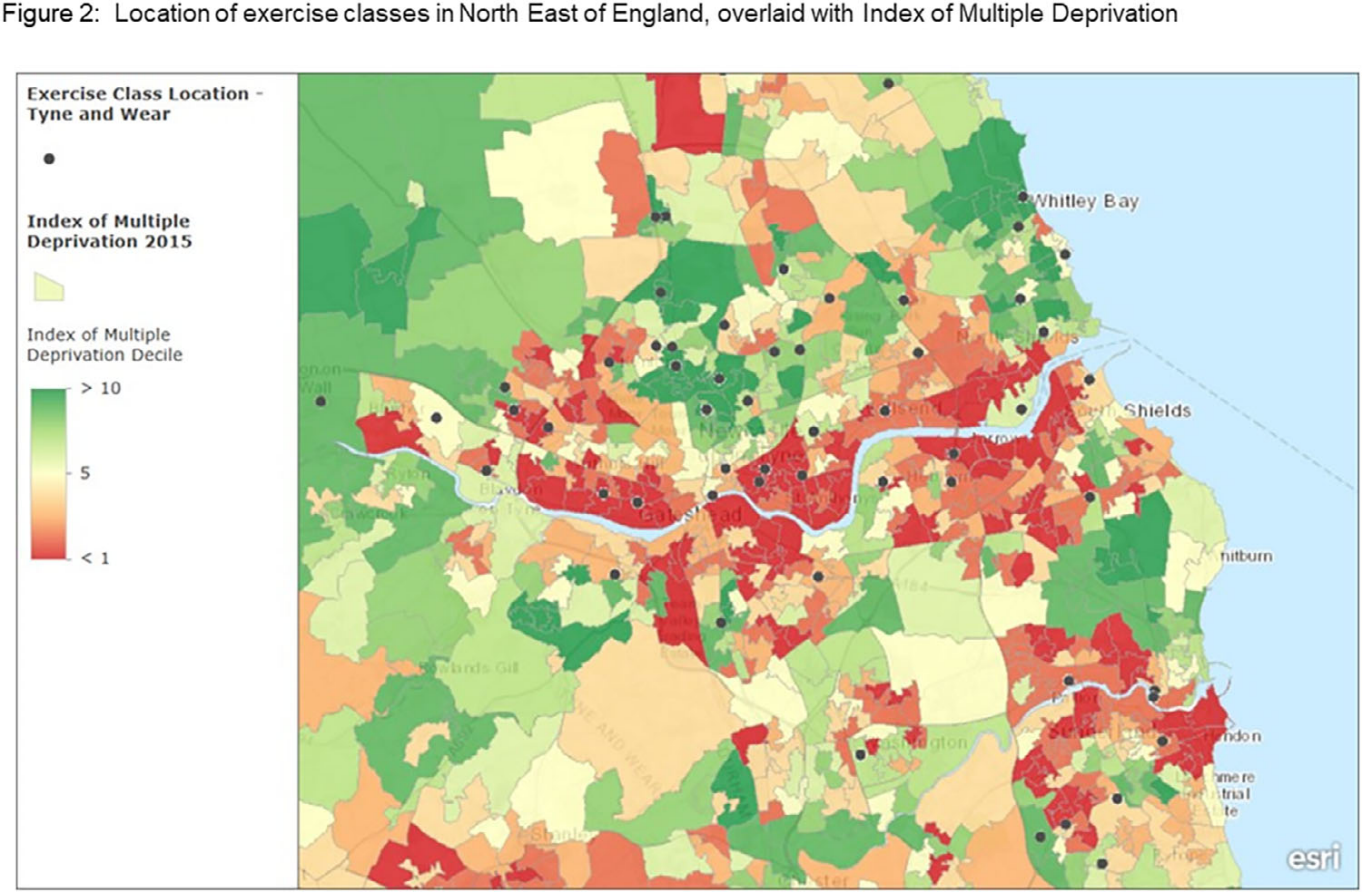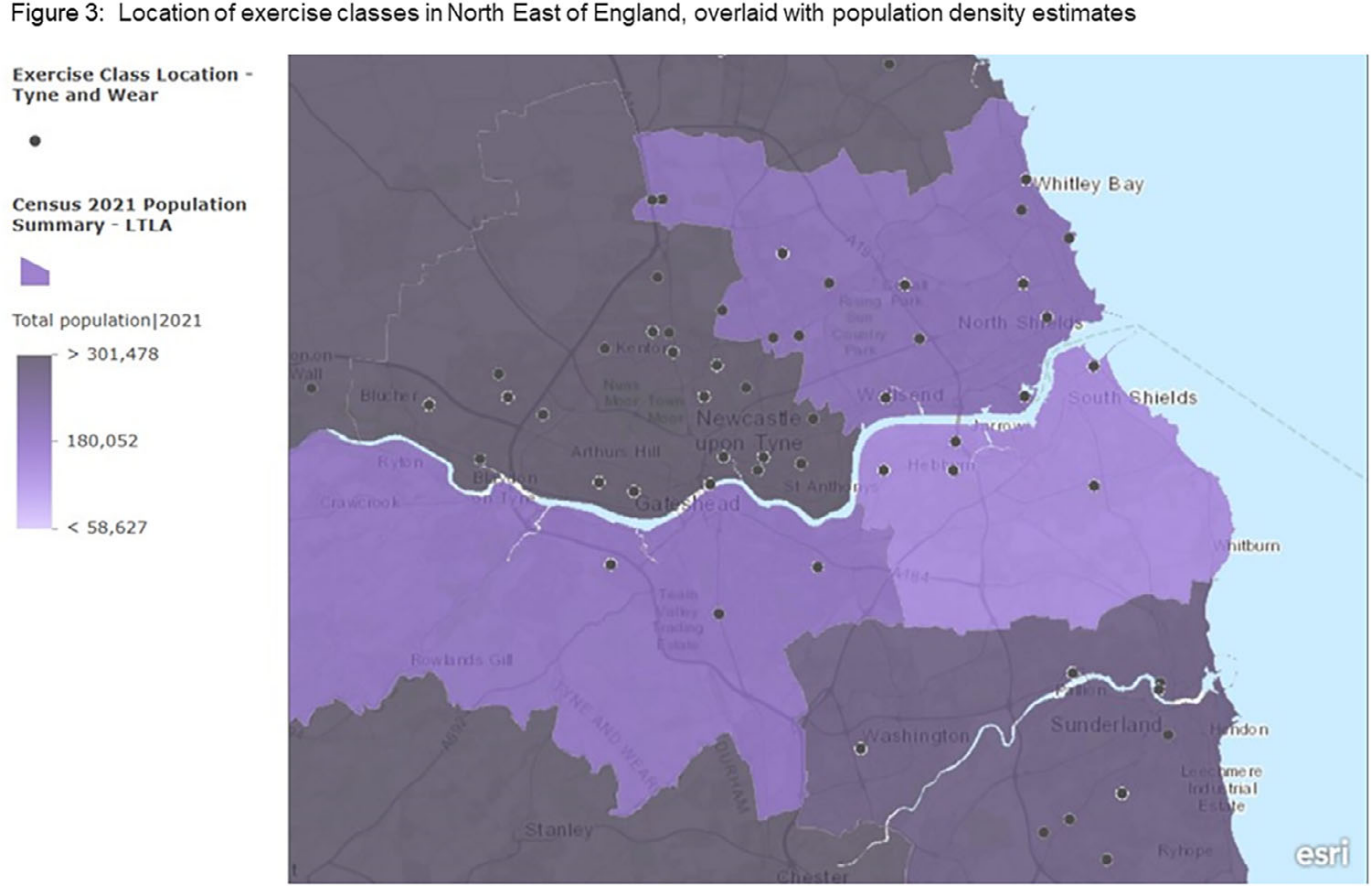# Are exercise classes for older people, including those with dementia, located in areas with the greatest need in the North East of England?

**DOI:** 10.1002/alz.092226

**Published:** 2025-01-09

**Authors:** Ríona Mc Ardle, Lucy Foster, Annabelle Long, Lisa Alcock

**Affiliations:** ^1^ Newcastle University, Newcastle Upon Tyne United Kingdom; ^2^ Newcastle University, Newcastle, Tyne and Wear United Kingdom; ^3^ University of Nottingham, Nottingham, Nottinghamshire United Kingdom

## Abstract

**Background:**

Approximately 944,000 people are living with dementia in the UK (∼0.8% of the population). The World Health Organisation consider dementia a public health priority. Efforts to prevent dementia and to decelerate its progression to dependency/disability are vital.

Promoting physical activity (e.g. exercise classes) can support dementia prevention and once diagnosed, support independence. Previous research indicates that most exercise classes for older adults in the East Midlands of England are in areas with small percentages of older inhabitants and only 2% are cited as suitable for dementia. As older adults in the North East (NE) of England have worse health outcomes, greater socioeconomic deprivation and lower life expectancy, we aimed to identify the geographical distribution and population characteristics of exercise classes in NE of England.

**Method:**

For preliminary analysis, one county in NE of England was selected, Tyne and Wear, an area of 538km^2^ with an estimated population of 1,136,371 (19% aged ≥65 years) in 2021. The area is primarily urban (∼98%). Exercise classes were identified via a comprehensive Google search undertaken in December 2023 and geocoded. Data were input into a geographic information system (ArcGIS, Esri). Spatial analysis tools in ArcGIS calculated number and percentage of classes with respect to population age, socio‐economic status and population density.

**Result:**

427 exercise classes were identified as suitable for older adults; <1% (n = 2) were advertised as appropriate for people with dementia. 5.4% were situated in areas where the predominant age group are aged ≥65years (Fig. 1), 20.6% in areas with the greatest socio‐economic deprivation (Fig. 2; based on Index of Multiple Deprivation) and 98% in urban areas (Fig. 3).

**Conclusion:**

Results highlight that there are limited opportunities for people with dementia to participate in exercise classes in Tyne and Wear, England. Additionally, most exercise classes are in urban areas where a low percentage of the population are aged ≥65years, limiting their accessibility to older people. Addressing this issue may contribute to health equity and disability‐free life expectancy in NE, supporting both dementia prevention and dementia post‐diagnostic support. Further work will consider the rest of NE of England (Northumberland, Durham) for a more representative analysis.